# Prevalence and associated factors of pickleball-related injuries among Korean recreational players: a multi-tournament cross-sectional study

**DOI:** 10.3389/fpubh.2026.1898734

**Published:** 2026-07-22

**Authors:** Kang-Jun Lee, Boseok Jeong, Seung-Hee Nam, Rokbit S. Lee, Jinmoo Heo, Kyung-Min Kim

**Affiliations:** 1 Department of Sport Science, Sungkyunkwan University, Suwon-si, Republic of Korea; 2 Department of Sport Industry Studies, Yonsei University, Seoul, Republic of Korea

**Keywords:** epidemiology, injury prevention, racket sports, recreational athletes, sports injury

## Abstract

**Background:**

Pickleball participation has grown rapidly in South Korea, yet epidemiological data on injuries in Korean players remain limited. This study aimed to describe the 12-month prevalence and characteristics of pickleball-related injuries and to identify factors associated with injury occurrence in a multi-tournament sample of Korean recreational players.

**Methods:**

A cross-sectional survey was administered on-site at multiple national pickleball tournaments held in South Korea in 2025. Eligible participants (*n* = 383; 249 men, 134 women; mean age 47.0 ± 12.8 years) completed a structured questionnaire assessing demographics, playing characteristics, and self-reported musculoskeletal injuries during the preceding 12 months. Injury characteristics including body region, tissue type, onset mechanism, and duration of activity cessation were described. Logistic regression analysis was performed to identify factors associated with injury occurrence, with odds ratios (ORs) and 95% confidence intervals (CI) reported.

**Results:**

Twelve-month injury prevalence was 38.1% (*n* = 146). Lower extremity injuries were most common (44.0%), with the knee (21.4%) and ankle/foot (15.8%) as the most frequent sites. Muscle and tendon injuries predominated (43.3%), followed by joint injuries (29.1%). Overuse injuries (52.1%) were slightly more frequent than traumatic injuries (47.9%). More than one-third of injured players reported at least 2 weeks of activity cessation. Logistic regression identified male sex (OR = 1.74, 95% CI: 1.04–2.90, *p* = 0.035) and greater weekly playing hours (OR = 1.06, 95% CI: 1.02–1.11, *p* = 0.006) as significantly associated factors with injury occurrence.

**Conclusion:**

Approximately two in five Korean recreational pickleball players sustained an injury over 12 months, with lower extremity and overuse injuries predominating. Male sex and greater weekly playing volume were independently associated with injury occurrence, providing a basis for targeted prevention strategies in Korean pickleball populations.

## 1 Introduction

Pickleball is a racket sport that combines elements of tennis, badminton, and table tennis and is played with a solid paddle and a perforated polymer ball on a court that corresponds to the standard dimensions of a doubles badminton court and approximately one-quarter the size of a tennis court (1). First introduced in the United States in 1965, pickleball has undergone a remarkable transformation from a backyard leisure activity into a globally competitive sport with a rapidly expanding international footprint (2). In the United States, the Sports and Fitness Industry Association (SFIA) named pickleball the nation’s fastest-growing sport for three consecutive years, with participation growing by 51.8% from 2022 to 2023 and by a cumulative 223.5% over the preceding 3 years (2, 3). By 2023, an estimated 36.5 million Americans were actively participating in the sport (3, 4). This global surge is widely attributed to the sport’s short learning curve, relatively low cost of entry, and emphasis on social interaction and community engagement. These qualities make it especially attractive to middle-aged and older adults (5–7). This expansion has reached South Korea, where pickleball was introduced around 2016 and gained institutional momentum with the establishment of the Korea Pickleball Association (KPA) in 2018 (8, 9). Multiple annual pickleball tournaments are now established across the country, and by 2025, an estimated 100,000 recreational players were active in approximately 500 clubs nationwide (8, 9). Therefore, systematic epidemiological attention is needed to clarify the public health implications of pickleball-related injuries.

However, this rapid growth carries an inherent risk of rising sport-related injuries. National surveillance data from the United States illustrate this trend: pickleball-related emergency department (ED) visits increased nearly 19-fold between 2014 and 2023, from approximately 1,313 to 24,461 cases (10). Pickleball-related hospitalizations increased by 257% between 2020 and 2022 alone, with older patients facing disproportionately higher risks of admission (11). The economic burden associated with these injuries has been estimated at $250 to $500 million annually in the United States, highlighting the growing public health significance of pickleball-related injuries and prompting a substantial body of epidemiological research (12). To date, however, the available literature remains largely concentrated in North American emergency department settings. Studies using national surveillance data have consistently identified older adults as the most frequently injured group, with strains, sprains, and fractures as the predominant injury types (10, 13, 14). Emergency department surveillance is inherently limited to injuries severe enough to require acute care and does not capture overuse injuries and minor acute trauma that constitute a substantial portion of the overall injury burden in sport (13–15). A more complete assessment of injury burden requires methods that capture injuries beyond those requiring acute care, including gradual-onset overuse conditions and time-loss episodes (16). Participant-based self-report surveys are well-suited for this purpose and have been employed to characterize the broader injury landscape of pickleball. In a nationwide U.S. survey, Owoeye et al. (17) reported a 12-month injury prevalence of approximately 68.5%, with lower extremity injuries predominating. Among older adult tournament players, Kim et al. (18) identified a 12-month injury prevalence of 32.1%, with lower extremity and muscle and tendon injuries most prevalent. These findings suggest that self-report surveys can identify a substantial burden of injuries that may not be captured through ED-based surveillance alone. Beyond characterizing injury prevalence and patterns, identifying factors associated with injury occurrence is important for informing targeted prevention strategies. This is particularly relevant in pickleball, a sport played by a broad range of age groups with substantial participation from middle-aged and older adults. Prior studies have suggested that injury occurrence may be associated with demographic, physical, and participation-related factors, including age, sex, body mass index (BMI), weekly playing hours, playing experience (17, 18). Together, these findings underscore the value of participant-based survey methods for characterizing the full spectrum of pickleball-related injury burden; however, the applicability of North American findings to Korean players remains limited, given the distinct demographic profiles, play environments, and sport cultures that characterize this population.

The participant-based epidemiological approach has recently been applied in South Korea. Jeong et al. (8) conducted a cross-sectional survey during a national pickleball championship in 2024 and reported a 12-month injury prevalence of 34.2% among 232 recreational players, with muscle and tendon injuries as the predominant tissue type and the knee, elbow/forearm, and shoulder as the most frequently affected sites. Logistic regression identified higher skill level (OR = 0.79) and greater weekly playing hours (OR = 0.91) as significantly associated with a reduced likelihood of injury, while age and sex were not significant predictors. Despite these contributions, several findings from that study deviate notably from those reported in international research and warrant independent confirmation in a larger, more representative sample. First, the inverse association between weekly playing hours and injury risk observed by Jeong et al. (8) (OR = 0.92) contrasts directly with the positive associations reported by Kim et al. (18) (OR = 1.06) and Owoeye et al. (17) (OR = 1.45) in US-based cohorts. This raises the question of whether this discrepancy reflects a genuine characteristic of Korean recreational players or a methodological artifact of single-site sampling. Second, overuse injuries accounted for 78% of all reported cases in Jeong et al. (8) —substantially exceeding the approximately 50% overuse proportion observed in broader recreational player surveys—and upper extremity injuries predominated over lower extremity injuries, a distribution that differs from the lower-extremity predominance consistently reported in North American pickleball cohorts and related racket sports (14, 17, 18). Third, BMI was not included as a predictor despite its documented relevance to injury risk in racket sports, and the backward stepwise regression approach employed may yield unstable risk factor estimates with a relatively modest analytic sample (n = 232), as predictor selection via stepwise elimination is sensitive to sampling variability. These methodological constraints limit the confidence with which findings can be generalized to the broader Korean recreational pickleball community and used to guide evidence-based prevention strategies. To address these gaps and evaluate the replicability of prior findings in a more representative sample, the present study pooled data from multiple national tournament events held across South Korea in 2025. This multi-tournament design provides a larger, more demographically diverse sample, enabling more robust estimation of 12-month injury prevalence and characteristics.

## 2 Materials and methods

### 2.1 Participants

This study employed a cross-sectional observational design. Data were collected on site from three pickleball tournaments held in South Korea between April and October 2025. Inclusion criteria were (1) age 18 years or older, (2) native Korean speaker, and (3) participation in at least one of the tournaments. Exclusion criteria were (1) pre-existing injuries unrelated to pickleball, and (2) refusal or withdrawal of consent. To avoid duplicate observation, participants who had already completed the questionnaire at an earlier tournament were not surveyed again at subsequent tournaments. Accordingly, the final dataset included only one completed questionnaire per participant. All participants received detailed information about the study’s objectives and procedures and provided written informed consents prior to data collection. Participants completed the questionnaire during the tournament period, either before or after matches. It took about 10–15 min for the participants to complete the questionnaire. To ensure confidentiality, survey responses were collected anonymously. This study was approved by the Institutional Review Board (IRB) of Sungkyunkwan University (IRB No. SKKU 2025–03-039).

### 2.2 Questionnaire

Data were collected using a structured questionnaire adapted from prior research in recreational pickleball populations (8, 18). The questionnaire comprised demographics, playing characteristics, and self-reported injuries within the past 12 months. Demographics included age, sex, and BMI, which was calculated by dividing body weight (kg) by height squared (m^2^). Playing characteristics included self-reported skill level from Korea Pickleball Association classification adapted from the USA Pickleball Association (19, 20). Skill level was categorized as below 3.0, 3.0, 3.5, over 4.0, with a “Do not know” option for participants who were uncertain of their level. In this rating system, skill level reflects current playing ability. In general, ratings below 3.0 indicate beginner-level players, 3.0 indicates advanced beginners, 3.5 indicates intermediate players, and ratings of 4.0 or higher indicate advanced intermediate or higher-level players (19, 20). Participants also reported their weekly play hours and length of playing experience. Additionally, participants were asked to report whether they had sustained any injuries related to pickleball during the past 12 months. Injuries were defined as any musculoskeletal complaint or physical harm regardless of whether formal diagnosis was made (21, 22). For those who reported injuries, additional details were collected on the affected body region using a modified Nordic body chart (23), type of injury (e.g., muscle/tendon, joint), classification as either traumatic (resulting from a specific identifiable event) or overuse (resulting from gradual onset), and duration of activity cessation due to injury. Because multiple responses were allowed for injury-related items, the total number of responses may differ across items.

### 2.3 Statistical analysis

All continuous variables were described as means and standard deviations (mean ± SD), whereas categorical variables were expressed as frequencies (n) and percentages (%). For multiple-response items, analyses were performed based on the total number of responses rather than the number of participants. Missing data were not imputed, and descriptive analyses were conducted using the available valid responses for each item. A logistic regression analysis was conducted to identify factors associated with the history of pickleball injuries. The demographic factors (age, sex, BMI) and play characteristics (skill level, the length of playing experience, and weekly play hours) were introduced into the regression model. Skill level was converted to numerical codes according to their ordinal variable coded in ascending order (24). Participants who selected “do not know” for skill level were excluded from the regression analysis. Missing data for other variables included in the regression model were handled using listwise deletion. Multicollinearity was assessed using variance inflation factor (VIF) values, with VIF > 5 considered indicative of high multicollinearity (25). Model fit was evaluated using the likelihood-ratio (omnibus) test comparing the full model with the null model and the Hosmer-Lemeshow goodness-of-fit test. Additionally, odds ratios (ORs) with 95% confidence interval (CI) were calculated to quantify the direction and strength of the associations, with ORs greater than 1 indicating increased odds of injury and ORs less than 1 indicating decreased odds of injury (26). All statistical analyses were conducted using R software, with a significance level set at *p* < 0.05 (version 4.3.1; R Foundation for Statistical Computing, Vienna, Austria).

## 3 Results

### 3.1 Demographic and play characteristics

Table 1 presents the demographics and playing characteristics of the participants. Overall, the sample was predominantly male and consisted mainly of middle-aged recreational players, with a mean BMI within the overweight range according to the Korean Society for the Study of Obesity criteria (27). Skill levels were concentrated at or below 3.0, indicating that many participants were beginner to lower-intermediate players. In addition, participants played approximately 9 h per week and had less than 2 years of pickleball experience.

**Table 1:** Descriptive summary of demographics and play characteristics.

Characteristics	Overall (*n* = 383)	Male (*n* = 249)	Female (*n* = 134)
Demographics			
Age, yrs	47.0 ± 12.8	47.5 ± 12.5	46.1 ± 13.3
BMI, kg/m^2^	23.8 ± 3.2	24.8 ± 3.0	22.1 ± 2.8
Play characteristics			
Skill level, *n* (%)			
Do not know	37 (9.7)	23 (6.0)	14 (3.7)
Under 3	105 (27.4)	63 (16.4)	42 (11.0)
3.0	130 (33.9)	84 (21.9)	46 (12.0)
3.5	69 (18.0)	44 (11.5)	25 (6.5)
Over 4	42 (11.0)	35 (9.2)	7 (1.8)
Weekly play hours, hrs	9.04 ± 5.4	8.96 ± 5.2	9.19 ± 5.9
Play experience, mo	18.3 ± 18.0	18.1 ± 18.6	18.6 ± 16.9

### 3.2 Self-reported injury prevalence and characteristics

Table 2 summarizes self-reported injury experience, injury mechanism, and time loss due to injury during the previous 12 months. Among all participants, 38.1% (n = 146) reported at least one pickleball-related injury during the previous year. Overuse injuries were slightly more common than traumatic injuries, with 6 more overuse responses. Regarding time loss due to injury, nearly one-third of injured participants reported at least 2 weeks of play cessation because of their injuries.

**Table 2:** Descriptive summary of self-reported injuries.

Characteristics	Overall (*n* = 383)	Male (*n* = 249)	Female (*n* = 134)
Injury-related outcomes			
Injury experience, *n* (%)			
YES	146 (38.1)	103 (26.9)	43 (11.2)
NO	237 (61.9)	146 (38.1)	91 (23.8)
Injury onset^a,b^, *n* (%)	144 (100)	103 (71.5)	41 (28.5)
Traumatic	69 (47.9)	44 (30.5)	25 (17.4)
Overuse	75 (52.1)	59 (41.0)	16 (11.1)
Playtime lost due to injury^a,b^, *n* (%)	142 (100)	100 (70.4)	42 (29.6)
1–2 days	23 (16.2)	18 (12.7)	5 (3.5)
Less than 1 week	35 (24.7)	27 (19.0)	8 (5.7)
1–2 weeks	33 (23.2)	23 (16.2)	10 (7.0)
2–4 weeks	27 (19.0)	17 (12.0)	10 (7.0)
1 month or more	24 (16.9)	15 (10.5)	9 (6.4)

^a^Items were assessed only among participants who reported at least one pickleball-related injury during the previous 12 months.

^b^There are missing data.

**Figure 1:**
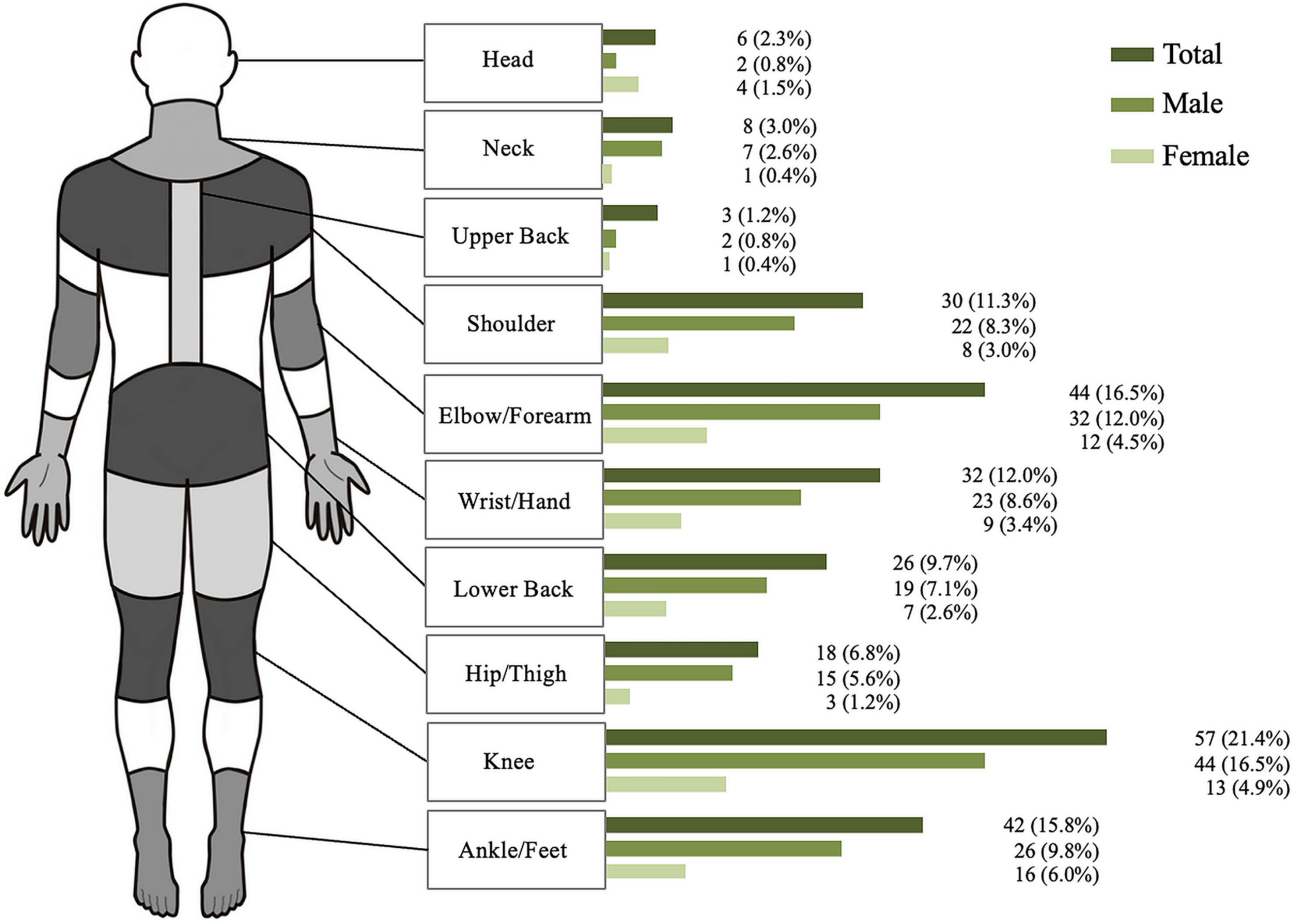
Self-reported pickleball-related injuries by body part from the 2025 Korean pickleball tournaments. Percentages were calculated using 266 valid body-part responses as the denominator. Male and female percentages indicate their contribution to the overall distribution. Body part was assessed as a multiple-response item. The body diagram was adapted from Jeong et al. (8), distributed under the terms of the Creative Commons Attribution License (CC BY). The injury data, bars, labels, and percentages were newly created using data from the present study.

Figure 1 demonstates the distribution of injured body parts based on 266 valid body-part responses. Because participants could report more than one injured body part, percentages were calculated using the total number of valid body-part responses rather than the number of injured participants. Lower extremity injuries (44.0%) were more common than upper extremity injuries (39.8%). The knee and ankle/foot were the most frequently reported lower extremity sites, whereas the elbow/forearm and wrist/hand were the most frequently reported upper extremity sites.

**Figure 2:**
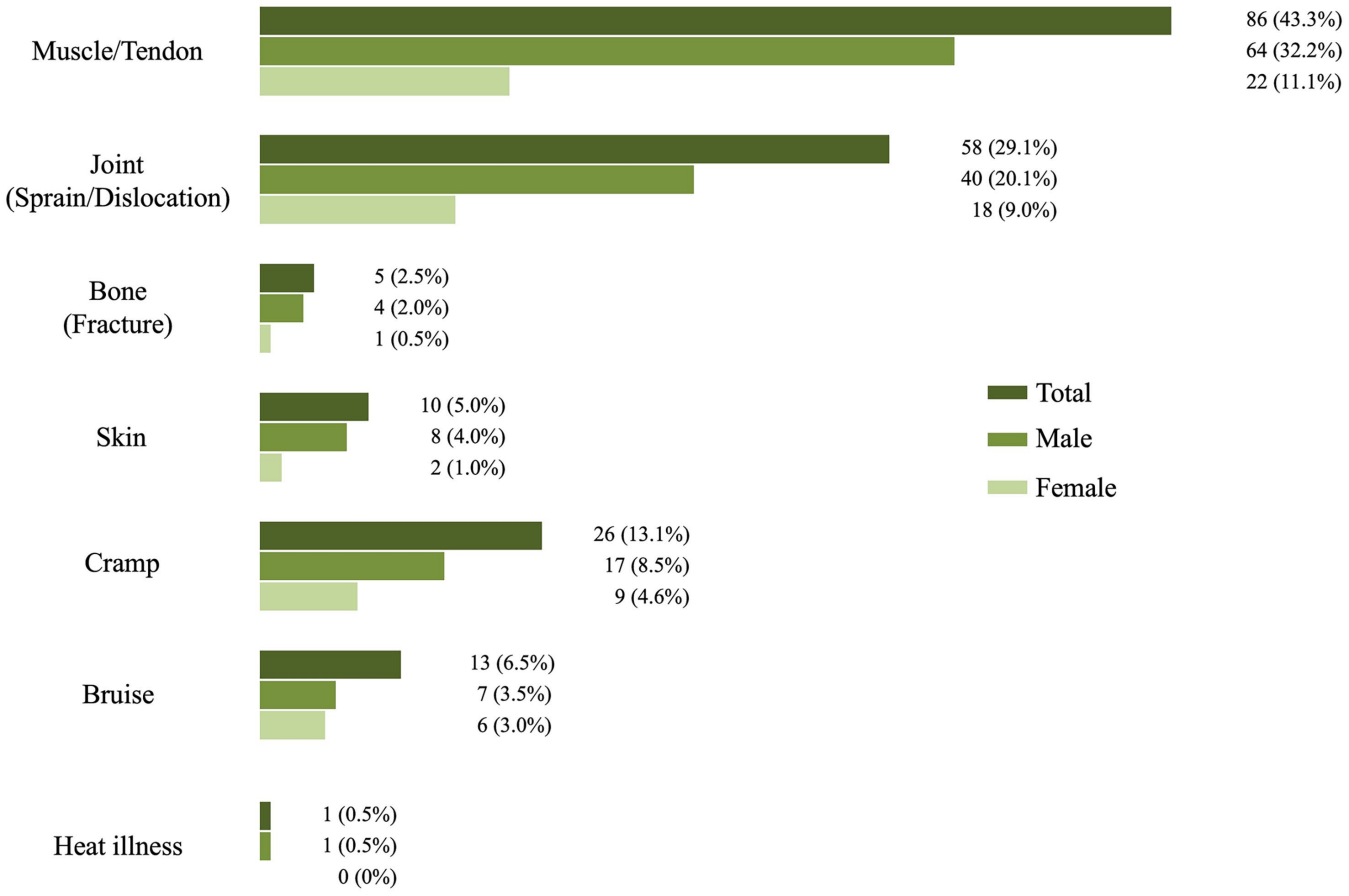
Self-reported pickleball-related injuries by tissue type from the 2025 Korean pickleball tournaments. Percentages were calculated using 199 valid tissue-type responses as the denominator. Male and female percentages indicate their contribution to the overall distribution. Tissue type was assessed as a multiple-response item.

Figure 2 shows the distribution of affected tissue types based on 199 valid tissue-type responses. Injury region and tissue type were analyzed as separate injury-characteristic items, and the total number of valid responses differed across these items. For example, the same tissue type, such as muscle/tendon, could be reported for different injured body parts, such as the knee and ankle/foot, indicating that body-part and tissue-type responses did not necessarily correspond one-to-one. Muscle/tendon injuries were the most frequently reported tissue type, followed by joint injuries, including sprains and dislocations. Together, these two categories accounted for the majority of affected tissue-type responses, whereas cramp, bruise, skin injury, bone injury, and heat illness were less frequently reported.

### 3.3 Factors associated with the pickleball-related injuries

Table 3 summarizes the logistic regression analysis of factors associated with pickleball-related injuries. The logistic regression analysis included 346 participants after excluding 37 participants who selected “do not know” for skill level and no participants with missing data on other variables. All VIF were below 5 (1.09–1.38), indicating no evidence of multicollinearity. The overall model showed borderline improvement over the null model, *χ*^2^(6) = 12.11, *p* = 0.060. The Hosmer-Lemeshow test indicated no evidence of poor model fit (*χ*^2^(8) = 5.15, *p* = 0.742). Despite the borderline omnibus test, sex and weekly play hours emerged as significant associated factors with higher odds of reporting a pickleball-related injury. Male players had significantly greater odds of reporting an injury than female players (B = 0.553, SE = 0.262, *p* = 0.035). Specifically, the odds ratio was 1.74 (95% CI, 1.04–2.90), indicating that the odds of injury were 74% higher in male players than in female players. Weekly play hours was also a significant factor (B = 0.062, SE = 0.022, *p* = 0.006). The odds ratio was 1.06 (95% CI, 1.02–1.11), suggesting that each additional hour of weekly play was associated with a 6% increase in the odds of injury. In contrast, age, BMI, skill level, and playing experience were not significantly associated with injury in the model (*p* > 0.05).

**Table 3:** Logistic regression analyses of factors associated with the pickleball-related injury.

Variables	*χ*^2^ (df)	Δ*χ*^2^ (df)	B	SE B	Wald	df	*p*-value	Odds ratio	95% CI for odds ratio
Intercept	12.11 (6)	–	0.777	1.013	0.006	1	0.939	1.08	(0.15, 7.87)
Age			−0.004	0.009	0.180	1	0.671	1.00	(0.98, 1.02)
Male			0.553	0.262	4.452	1	0.035*	1.74	(1.04, 2.90)
Body mass index			−0.045	0.039	1.341	1	0.247	0.96	(0.89, 1.03)
Skill level			−0.068	0.120	0.318	1	0.573	0.93	(0.74, 1.18)
Length of playing experience			0.003	0.007	0.125	1	0.724	1.00	(0.99, 1.02)
Weekly play hours			0.062	0.022	7.639	1	0.006**	1.06	(1.02, 1.11)

*p < 0.05, **p < 0.01.

## 4 Discussion

### 4.1 Prevalence and characteristics of pickleball injuries

The present study found that 38.1% of Korean recreational pickleball players reported at least one pickleball-related injury during the previous 12 months. This prevalence is broadly comparable to the 34.2% reported in a previous Korean self-report study by Jeong et al. (8) and the 32.1% reported by Kim et al. (18) among older players at the US Open Pickleball Championship. In contrast, Owoeye et al. (17) reported a substantially higher annual injury prevalence of 68.5% in a nationwide sample of pickleball players. These differences may be partly explained by variation in sampling frame, participant characteristics, and injury definition. Tournament-based studies may preferentially include players who are sufficiently active and healthy to compete, whereas nationwide surveys may capture a broader range of recreational players, including those with persistent symptoms or limited competition participation. Therefore, the relatively lower estimates observed in tournament-based studies should not be interpreted as evidence of a lower overall injury burden, but rather as reflecting methodological differences across studies. Despite these methodological considerations, the present study provides additional epidemiological evidence on pickleball-related injuries among Korean recreational players.

Regarding injury region, lower extremity injuries accounted for a slightly larger proportion of reported injuries than upper extremity injuries in the present study. Although the distribution of injured body regions has varied across previous pickleball studies, lower extremity involvement has been frequently reported, including in studies of US Open Pickleball Championship players (57.9%) and a nationwide sample of pickleball players (57.0%) (17, 18). It is noteworthy, however, that a prior cross-sectional survey of Korean recreational pickleball players reported the opposite distribution, with upper extremity injuries (44.8%) predominating over lower extremity injuries (42.3%) (8). This reversal may partly reflect differences in skill level composition between the two samples: the present study included a higher proportion of players rated above skill level 4.0 (11.0% vs. 3.0% in Jeong et al.) (8), and more technically proficient players tend to engage in greater dynamic court movement placing proportionally higher mechanical demand on the lower extremities whereas less experienced players may sustain more upper extremity injuries from suboptimal swing mechanics (8). These observations suggest that the distribution of injured body regions in Korean recreational pickleball players may be sensitive to the skill level composition of the sample, and highlight the value of multi-site, multi-tournament sampling for producing more representative injury region estimates. The relatively frequent involvement of the lower extremities in the present study may be explained by the biomechanical demands of pickleball, including rapid directional change, lunging, jumping, acceleration and deceleration, and lateral movement, which place repeated stress on the lower extremities such as the knee and ankle (14, 28, 29). This interpretation is further supported by similar injury patterns observed in other racket sports with comparable movement demands, such as tennis and badminton, where lower extremity injuries accounted for 41.7 and 44.4% of reported injuries, respectively (30, 31). These findings suggest that lower-extremity injury patterns in pickleball may be partly attributable to repetitive multidirectional movement demands shared across racket sports.

In terms of tissue types, muscle/tendon injuries were the most reported in the present study (43.3%), followed by joint injuries (29.1%). This pattern is consistent with previous pickleball research. Jeong et al. (8) reported that muscle/tendon injuries were the most common tissue type (33.3%), followed by joint injuries (28.3%), while Kim et al. (18) showed high proportions of muscle/tendon injuries (61.8%) and joint injuries (44.3%). Although pickleball-specific biomechanical evidence explaining this tissue distribution remains limited, biomechanics studies in racket sports suggest that sport-specific striking and court-movement tasks place mechanical loads on muscle-tendon and joint structures (32–34). Given that pickleball shares some biomechanical characteristics with racket sports, the frequent reporting of muscle/tendon and joint-related injuries may reflect similar repetitive loading patterns.

In the present study, overuse and traumatic injuries occurred in relatively similar proportions, indicating that overuse injuries accounted for a substantial proportion of reported injuries. This pattern differs from emergency-based studies, which primarily capture acute traumatic injuries requiring medical attention, such as falls, sprains, and fractures (13, 14). In contrast, self-reported surveys of recreational players may be more sensitive to non-emergency and gradual-onset injuries. Such injuries may reflect cumulative sport-related exposure, in which symptoms develop gradually rather than following a single identifiable traumatic event (32, 35, 36). This may help explain why previous self-report studies in pickleball and tennis have reported relatively high proportions of overuse or gradual-onset injuries. For example, a previous Korean pickleball study reported that 78% of injuries were overuse-related, while tennis studies reported gradual-onset or overuse injury proportions of 69.6% (8, 37). Thus, the present findings suggest that self-reported injury surveillance may capture a broader spectrum of pickleball-related musculoskeletal problems than emergency department-based data, including gradual-onset injuries that may not require urgent medical care.

Furthermore, the present study assessed time loss due to injury and found that more than one-third of injured players reported at least 2 weeks of play cessation. This finding suggests that pickleball-related injuries may meaningfully disrupt continued participation, even when they may not require emergency medical care. A similar pattern was reported in a previous study of Korean pickleball players, in which 37.9% of injured participants reported more than 2 weeks of play cessation (8). Kim et al. (18) also reported that a notable proportion of US Open Pickleball Championship players experienced prolonged play cessation, with 21.1% stopping play for 2–4 weeks and 28.9% stopping for more than 1 month. Given the injury profile observed in the present study, the reported time loss may be partly understood in relation to the types of injuries commonly experienced by participants. However, no pickleball study has examined whether time loss differs according to injury region or tissue type. This represents a remaining gap because descriptive research in other racket sports suggests that time-loss duration may vary by injury location and structure. For example, Lambert et al. (38) reported that the longest time-loss injuries were an unspecified shoulder injury in tennis, a foot-ligament injury in badminton, and a knee injury in table tennis. Therefore, future pickleball studies should examine whether specific injury regions or tissue types are associated with longer play cessation.

### 4.2 Factors associated with the pickleball-related injuries

Male players had significantly greater odds of sustaining a pickleball-related injury compared to female players (OR = 1.74, 95% CI: 1.04–2.90). This finding aligns with epidemiological evidence from international pickleball cohorts. In the United States, Owoeye et al. (17) documented male sex as a significant predictor of injury (OR = 1.33, 95% CI: 1.07–1.65), and Kim et al. (18) similarly reported that female tournament participants had significantly lower odds of injury compared to their male counterparts (OR = 0.42, 95% CI: 0.21–0.87), suggesting that male sex may represent a potential factor associated with pickleball-related injury across different settings. National emergency department surveillance data further support this disparity, indicating that male players exhibit higher rates of overexertion and acute trauma during play (10). While a prior domestic study by Jeong et al. (8) reported that sex was not a significantly associated with injury occurrence among Korean tournament participants, the discrepancy between their findings and the present results may reflect differences in study design and sample composition. In particular, the present study included participants from multiple tournaments and a wider range of competitive levels, which may have captured greater variability in player characteristics and playing exposure. Beyond these methodological differences, the higher injury odds observed among male players may be partly related to differences in playing patterns or sport-specific movement demands (33). However, because these factors were not directly assessed in the present study, this interpretation should be made cautiously.

In addition, higher weekly playing hours emerged as a significant factor of injury, with each additional hour of play per week associated with a 6% increase in injury odds (OR = 1.06, 95% CI: 1.02–1.11). This finding contrasts with a prior Korean pickleball study by Jeong et al. (8), which reported an inverse association between weekly playing hours and injury odds. However, the present result is consistent with previous U. S. pickleball studies, including Kim et al. (18), who reported a similar association for weekly play hours (OR = 1.06, 95% CI: 1.00–1.12), and Owoeye et al. (17), who found higher injury odds among players participating three or more times per week (OR = 1.45). Thus, although pickleball-specific findings are not entirely consistent, the association between greater weekly playing volume and injury risk is supported by evidence from related racket and paddle sports. For example, tennis players who trained more than 4.5 h per week were reported to have more than twice the odds of sustaining a musculoskeletal injury (OR = 2.04, 95% CI: 1.16–3.60) (39), and players engaging in more than 3–6 h per week face elevated risk of overuse injuries (39–41). From an exposure standpoint, accumulated repetitive mechanical loading and fatigue may contribute to skeletal muscle damage (42). In addition, prolonged intermittent play has been shown to reduce neuromuscular function during match play, which may impair movement control under fatigue and thereby increase susceptibility to repetitive overload and some fatigue-related traumatic injury events (43, 44). These mechanisms provide a plausible explanation for the association between greater weekly playing volume and injury occurrence in the present study. However, the magnitude of this association should be interpreted cautiously; although statistically significant, the OR of 1.06 indicates only a modest increase in injury odds per additional hour of weekly play. Nevertheless, weekly playing hours may be a practical exposure-related factor to consider when advising recreational pickleball players.

In contrast to the significant contributions of sex and weekly playing hours, age, BMI, skill level, and playing experience were not independently associated with injury risk in the multivariate model (*p* > 0.05). The absence of these associations may be explained by several sample-specific factors. Regarding age, Owoeye et al. (17) reported that age was a significant predictor across multiple age groups (ORs 1.83–3.11) in a sample with a considerably older mean age of 62.7 years. The present sample, with a mean age of 46.2 years, may not have included sufficient proportions of older adults among whom age-related physiological declines such as reduced bone density and diminished neuromuscular control are most pronounced, potentially attenuating the observed association between age and injury occurrence (45). Similarly, the non-significance of BMI may reflect the age composition of our sample. The association between lower BMI and injury identified by Kim et al. (18) was observed in an exclusively older adult population, where lower BMI more likely reflects age-related muscle mass loss and reduced physical reserve. In the present mixed-age sample, a mean BMI of 23.8 kg/m^2^ was close to the overweight cutoff according to Korean criteria (27) and may not carry the same physiological implications. Regarding skill level and playing experience, the distributional characteristics of the present sample may have limited the ability to detect significant associations. Most participants (61.3%) were concentrated at or below skill level 3.0, with only 11.0% reporting a level above 4.0. This restricted distribution may have reduced the variability needed to observe an association between higher skill level and injury occurrence, such as the protective association reported by Jeong et al. (OR = 0.789) (8). Similarly, playing experience was relatively short in the present study, with a mean of 18.3 months. This was shorter than that reported in a previous U. S. pickleball study, in which participants had an average playing experience of 43.8 months (46). The relatively limited playing experience in the present sample may have reduced the contrast between short- and long-term players, thereby limiting the ability to detect experience-related differences in injury risk. This interpretation is supported by Owoeye et al. (17), who reported that players with more than 5 years of pickleball experience had higher injury odds (OR = 1.50), suggesting that longer-term participation may reflect greater accumulated exposure to pickleball-specific physical demands. Therefore, the non-significant associations observed for age, BMI, skill level, and playing experience should be interpreted within the context of the present sample characteristics and the distribution of these variables.

### 4.3 Clinical implications

The findings of this study have implications for clinical practice and player safety in recreational pickleball. Given the frequent occurrence of lower-extremity, muscle/tendon, joint-related, and overuse injuries, prevention efforts may need to consider physical conditioning, movement quality, and adequate recovery to support safe participation. Within physical conditioning, strength and neuromuscular training may be useful for improving load tolerance, movement control, and physical preparedness for the repetitive and multidirectional demands of pickleball, based on broader sports injury-prevention evidence (47, 48). In addition, the finding that more than one-third of injured players reported at least 2 weeks of play cessation suggests that clinicians should consider not only injury occurrence, but also recovery duration and return-to-play guidance when managing recreational pickleball players. Beyond these injury pattern findings, the findings on factors associated with the injury occurrence may also provide clinically relevant information for player education and counseling. Although male players showed higher odds of injury, sex is a non-modifiable factor and may be more useful as a risk marker than as a direct prevention target. More practically, the positive association between weekly play hours and injury odds suggests that playing volume should be considered when advising recreational pickleball players. Clinicians should consider discussing weekly playing volume during injury-prevention counseling, particularly among players who participate frequently or play for prolonged durations. Rather than recommending general activity restriction, guidance should focus on load management, including progressive increases in weekly play time, scheduled recovery periods, and modification of play volume.

### 4.4 Limitations and recommendations for future research

This study has several limitations. First, although this study included participants from multiple tournaments, the sample was still limited to tournament participants, which may have introduced selection bias. Therefore, the findings may not be fully generalizable to non-tournament recreational pickleball players, beginners, or individuals who participate primarily in community-based settings. Future studies should include broader pickleball populations across both competitive and non-competitive settings to better understand injury patterns in the overall pickleball community. Second, injury history and playing characteristics were collected using self-reported retrospective questionnaires, which may have introduced recall bias. Participants may have inaccurately recalled the type, timing, or severity of injuries sustained during the previous 12 months, potentially affecting the reported prevalence and characteristics of pickleball-related injuries. In addition, injuries were not medically confirmed, which may have limited the accuracy of injury type and severity. Future studies incorporating prospective injury surveillance, clinician-confirmed injury classification, or standardized injury reporting systems would help improve the accuracy of injury identification and severity assessment. Third, the cross-sectional design enabled the collection of injury prevalence and playing characteristic data from a relatively large sample within a practical tournament-based setting. However, this design limits the ability to determine temporal or causal relationships between potential associated factors and injury occurrence. Furthermore, although male sex and weekly playing hours were significantly associated with injury occurrence, the overall regression model did not reach conventional statistical significance compared with the null model (*p* = 0.060). These associations should be interpreted with appropriate caution. Therefore, future prospective studies are needed to clarify whether factors such as weekly playing hours, sex, and other playing characteristics contribute to subsequent injury occurrence over time. Finally, this study could not fully characterize specific injury profiles because detailed contextual information was limited. Injury region, tissue type, injury mechanism, and time loss were analyzed as separate injury characteristic variables, and additional contextual information such as injury side, limb dominance, court surface, and specific injury-related movements was not collected. A more detailed profile-based analysis incorporating these factors may be clinically informative because the burden of injury may depend on the combination of injury characteristics and sport-specific contextual factors. Therefore, future studies should use integrated injury characteristic analyses to better clarify the severity and clinical relevance of specific pickleball injury patterns.

## 5 Conclusion

This study provides epidemiological evidence on the 12-month prevalence, characteristics, and associated factors of pickleball-related injuries among Korean recreational players using data from multiple tournaments held in 2025. The current study found that 38.1% of participants reported at least one pickleball-related injury during the previous 12 months, with lower-extremity, muscle/tendon, joint-related, and overuse injuries commonly reported. In addition, a substantial proportion of injured players experienced at least 2 weeks of play cessation, suggesting that pickleball-related injuries may meaningfully affect continued participation. Logistic regression analysis showed that male sex and greater weekly playing hours were significantly associated with higher odds of injury, whereas age, BMI, skill level, and length of playing experience were not significantly associated with injury history. These findings extend current knowledge of pickleball injury epidemiology in South Korea and provide practical information for understanding the injury burden and participation-related factors among recreational pickleball players. Future studies including broader pickleball populations beyond tournament participants, and more detailed injury characteristics analyses are needed to further validate and expand these findings.

## Data Availability

The raw data supporting the conclusions of this article will be made available by the authors, without undue reservation.
